# Smoking behaviour among adult patients presenting to health facilities in four provinces of Vietnam

**DOI:** 10.1186/s12889-021-10880-z

**Published:** 2021-05-01

**Authors:** Wan-Chun Huang, Ngoc Yen Pham, Thu Anh Nguyen, Van Giap Vu, Quy Chau Ngo, Viet Nhung Nguyen, Becky Freeman, Stephen Jan, Joel Negin, Guy B. Marks, Gregory J. Fox

**Affiliations:** 1 Woolcock Institute of Medical Research, Hanoi, Vietnam; 2 South Western Sydney Clinical School, University of New South Wales, Sydney, Australia; 3 Division of Thoracic Medicine, Department of Internal Medicine, Shuang Ho Hospital, Taipei Medical University, Taipei, Taiwan; 4 Respiratory Center, Bach Mai Hospital, Hanoi, Vietnam; 5 National Tuberculosis Control Program of Vietnam, Hanoi, Vietnam; 6 School of Public Health, University of Sydney, Sydney, Australia; 7 Health Economics and Process Evaluation Program, George Institute for Global Health, Sydney, Australia; 8 Faculty of Medicine and Health, University of Sydney, Sydney, Australia

**Keywords:** Smoking, Prevalence, Smoking cessation, Quit smoking, Health facility, Vietnam

## Abstract

**Background:**

Attendance at healthcare facilities provides an opportunity for smoking cessation interventions. However, the smoking behaviours of patients seeking healthcare in Vietnam are not well-understood. We aimed to evaluate behaviours related to smoking among patients presenting to health facilities in Vietnam.

**Methods:**

We conducted a cross-sectional study in 4 provinces of Vietnam. Consecutive patients aged ≥15 years presenting to 46 health facilities were assessed. Current smokers were randomly selected to complete a full survey about smoking behaviour, quit attempts, and preparedness to quit.

**Results:**

Among 11,245 patients who sought healthcare, the prevalence of current smoking was 18.6% (95% CI: 17.8–19.4%) overall, 34.6% (95% CI: 33.2–36.0%) among men and 1.1% (95% CI: 0.8–1.3%) among women. Current smokers who were asked about smoking by healthcare providers in the last 12 months were more likely to make quit attempts than those not asked (40.6% vs 31.8%, *p* = 0.017). Current smokers who attempted to quit in the past 12 months made limited use of cessation aids: counselling (1.9%) and nicotine replacement therapy (10%). A higher proportion of patients wanted to quit in the next month at national/provincial hospitals (30.3%) than those visiting district hospitals (11.3%, *p* < 0.001) and commune health centres (11.1%, *p* = 0.004).

**Conclusions:**

Smoking is common among male patients presenting to healthcare facilities in Vietnam. Formal smoking cessation supports are generally not used or offered. This population is likely to benefit from routine smoking cessation interventions that are integrated within the routine healthcare delivery system.

**Supplementary Information:**

The online version contains supplementary material available at 10.1186/s12889-021-10880-z.

## Background

Tobacco smoking remains the leading preventable risk factor for chronic disease and premature death in both developed and developing countries [1]. Reducing the prevalence of smoking is a high priority in global health [2].

Evidence-based strategies have been shown to reduce smoking prevalence in many settings. The MPOWER framework [3], endorsed by the World Health Organization (WHO), is a package intended to assist implementation of effective interventions. The O refers to offering help to quit tobacco use, such as quit advice from health professionals, cessation medications, and quit lines.

Health facilities provide a setting in which smokers may be amenable to smoking cessation efforts, as they often present with symptoms caused by smoking-related health conditions. Smoking cessation interventions are effective when tailored to patients in various healthcare settings, such as primary care, emergency room, and inpatient department [4]. The WHO, which coordinates the implementation of the Framework Convention on Tobacco Control, has also highlighted the importance of smoking cessation efforts in health care settings [5, 6].

Despite wide recognition of effectiveness and the promulgation of government policies, there is limited implementation of cessation programmes in many healthcare settings [7–10]. In Vietnam, the government enacted a comprehensive Law on Prevention and Control of Tobacco Harms in 2012. This was followed by the Vietnamese government’s Directive 05/CT-BYT that reinforces the delivery of cessation services within facilities at all levels of the healthcare system. However, there is little evidence about the extent to which smokers receive support to quit smoking during routine attendance at healthcare facilities.

A 2015 population-based survey found current smoking prevalence of 45.3% among males and 1.1% among females in Vietnam [11]. More than half of current smokers surveyed were considering quitting. The majority of those who attempted to quit in the past 12 months did not seek assistance. The prevalence of smoking among patients attending healthcare facilities, their preparedness to quit, and their access to effective smoking cessation interventions have not been well-characterised.

This study aimed to evaluate the behaviours related to smoking among patients seeking healthcare, including prevalence of smoking and past quit attempts. It also aimed to determine the attitudes towards quitting smoking among patients who were smokers.

## Methods

### Design and study setting

We performed a cross-sectional survey within 46 government health facilities selected from four Provinces of Vietnam. This Southeast Asian country is a middle-income country with a population of 96 million people. The public healthcare system is organised into four levels: central (national) hospitals, provincial hospitals, district hospitals and commune health centres. This study was undertaken in four of Vietnam’s 63 provinces, including two in the north of Vietnam (the capital, Hanoi, and Thanh Hoa Province) and two in the south (Ho Chi Minh City and Ca Mau Province). Participants were recruited from health facilities at all four levels of the health system in each province.

### Sampling of study sites

Major central and provincial hospitals in each province were included. In addition, four district hospitals were randomly selected in each province. Within each selected district, two commune health centres were also selected by random sampling. The probability of each facility being chosen was proportional to the populations of the districts and communes within which the health facilities were located. Within each central and provincial hospital, one department was selected by convenience sampling from among the wards or clinics in which patients with respiratory diseases were managed, or smokers were routinely assessed. At district hospitals, patients were recruited on outpatient clinics.

### Selection of study participants

Eligible patients were aged 15 years and over attending selected study sites. Patients were ineligible if they were unable to complete the survey due to substantial communication difficulties, lived in another province, or were known to be pregnant.

Study participants were selected at random from among the following groups of patients attending the selected healthcare facilities: (i) Consecutively presenting outpatients presenting with any medical condition (with a sampling fraction determined based upon the recruitment capacity of study staff); (ii) Consecutively presenting outpatients with one or more respiratory symptoms (dyspnoea, cough, wheezing, and/or chest tightness); and (iii) Inpatients with any medical condition at participating hospitals on the day of the survey. The age and gender of patients in each group were recorded in a registration book. From among patients listed in the registration book, a random sample was selected and invited to participate in the study.

All eligible participants selected to be included in the study were asked to give written informed consent. In order to assess potential selection bias, patients who declined to complete the full survey were asked to provide verbal consent and complete a “minimal data questionnaire” that included their age and gender.

### Questionnaire

Data collected for the full survey included age, gender, body weight, height, current and past smoking behaviours, current tobacco products, history of advice to quit smoking from healthcare providers, quit attempts in the last 12 months, smoking cessation services used in the last 12 months, and preparedness to stop smoking. Other details that were collected included past medical history, comorbidities, the highest level of educational attainment and current occupation. The questionnaire was developed based on published questionnaires [12, 13].

### Statistical methods

The prevalence of smoking and associated 95% confidence intervals were calculated from the proportion of all enumerated individuals presenting to health facilities who reported smoking within the preceding 30 days. Multiple imputation was used to impute missing values for smoking status, using age and gender as the observed data [14]. We separated males and females in the analysis of smoking prevalence, because the Global Adult Tobacco Survey (GATS) 2015 showed a significant disparity in the prevalence of smoking among males and females in the general population [11]. The standardised prevalence ratio was determined by comparing the differences in smoking rate among the study population and the general population, based upon population estimates from the GATS [11]. The confidence limits for the standardised prevalence ratio were obtained by bootstrapping. Comparisons were undertaken using chi-square test for categorical variables and analysis of variance for continuous variables. Analyses were conducted using SAS® (v9.4, SAS Institute, Cary Corp. NC. USA).

### Ethical issues

Ethical approval was provided by the Human Research Ethics Committee of the University of Sydney (2017/511), and the Institutional Review Board of the Bach Mai Hospital, Hanoi, Vietnam. Participants aged 18 and over provided written informed consent. Adolescents between 15 and 18 year of age provided verbal assent, and their parents provided written informed consent.

## Results

### Prevalence of smoking

Study participants were recruited between September 2017 and October 2018. Table 1 shows the prevalence of smoking by gender, age groups and levels of facility. Among 11,245 enumerated patients who visited health facilities during the observation period, the prevalence of current smoking was 18.6% (95% CI: 17.8–19.4%) overall, and 34.6% (95% CI: 33.2–36.0%) among men and 1.1% (95% CI: 0.8–1.3%) among women. Male patients aged 25 to 64 years were more likely to smoke than those younger than 25 years or older than 65 years. The prevalence among male patients visiting commune health centres (42.2%, CI: 36.7–47.7%), and district hospitals (39.3%, CI: 37.1–41.4%) was higher than that among patients visiting central/provincial hospitals (31.0%, CI: 29.2–32.8%). The prevalence among female patients was higher at commune health centres (4.4%, CI: 1.9–6.9) when compared to central/provincial hospitals (0.8%, CI: 0.5–1.2) and district hospitals (0.9%, CI: 0.5–1.3).

**Table 1:** Proportion of current smoking among patients presenting to health facilities, by age, sex and health system level

		All facilities (46 facilities) ***N*** = 9700				Central/provincial hospital (8 facilities) ***N*** = 4890				District hospital (16 facilities) ***N*** = 4287				Commune health centre (22 facilities) ***N*** = 523			
		Male ***N*** = 4620		Female ***N*** = 5080		Male ***N*** = 2373		Female ***N*** = 2517		Male ***N*** = 1956		Female ***N*** = 2331		Male ***N*** = 291		Female ***N*** = 232	
		n/N	% (95% CI)^**a**^	n/N	% (95% CI)^**a**^	n/N	% (95% CI)^**a**^	n/N	% (95% CI)^**a**^	n/N	% (95% CI)^**a**^	n/N	% (95% CI)^**a**^	n/N	% (95% CI)^**a**^	n/N	% (95% CI)^**a**^
**All age**		1595/4620	34.6 (33.2–36.0)	53/5080	1.1 (0.8–1.3)	699/2373	31.0 (29.2–32.8)	21/2517	0.8 (0.5–1.2)	771/1956	39.3 (37.1–41.4)	20/2331	0.9 (0.5–1.3)	125/291	42.2 (36.7–47.7)	12/232	4.4 (1.9–6.9)
**Age group (years)**	**15–24**	51/250	24.0 (18.3–29.6)	3/319	1.0 (− 0.1–2.1)	17/119	20.2 (12.3–28.1)	0/145	0	27/115	25.8 (17.6–34.0)	1/154	0.8 (− 0.7–2.2)	7/16	43.1 (18.3–67.8)	2/20	9.1 (−)^b^
	**25–34**	170/428	40.2 (35.8–44.7)	8/580	1.4 (0.5–2.4)	82/213	39.4 (33.3–45.5)	4/283	1.4 (0–2.8)	78/184	42.7 (35.6–49.7)	2/244	1.0(− 0.3–2.3)	10/31	32.8 (16.7–48.9)	2/53	3.6 (− 1.3–8.4)
	**35–44**	186/489	38.1 (33.8–42.4)	11/622	1.8 (0.7–2.8)	95/238	39.4 (33.5–45.4)	4/305	1.3 (−)^b^	79/201	39.1 (32.4–45.9)	6/293	2.0 (0.4–3.6)	12/50	24.9 (12.8–37.0)	1/24	3.5 (− 3.3–10.4)
	**45–54**	322/765	40.4 (37.0–43.9)	12/838	1.4 (0.6–2.2)	137/380	35.6 (31.1–40.1)	3/411	0.7 (− 0.1–1.6)	160/339	45.9 (40.6–51.3)	6/389	1.5 (0.3–2.7)	25/46	51.4 (37.5–65.3)	3/38	6.4 (− 0.6–13.5)
	**55–64**	495/1203	39.6 (37.0–42.2)	8/1218	0.7 (0.2–1.1)	206/586	34.7 (31.0–38.3)	5/552	0.9 (0.1–1.7)	253/553	45.3 (41.2–49.4)	3/618	0.5 (− 0.1–1.1)	36/64	54.4 (42.3–66.5)	0/48	1.8 (− 1.8–5.5)
	**65+**	371/1486	26.1 (24.0–28.3)	11/1503	0.7 (0.3–1.2)	162/837	22.5 (19.8–25.2)	5/821	0.6 (0.1–1.1)	174/564	30.1 (27.1–34.9)	2/633	0.4 (− 0.1–0.9)	35/84	40.7 (30.4–51.0)	4/49	7.0 (0.4–13.6)

^a^Pooled proportions. Missing values for smoking status were estimated for 1544 individuals using multiple imputation, with 95% confidence limits calculated based upon imputed values

^b^Between-imputation variance is zero

The standardised prevalence ratio of smoking among the population in the healthcare facilities was 0.80 (95% CI: 0.74–0.87) when compared with an age-matched sample from the general population reported in the GATS in 2015 [11]. When compared to a gender-matched sample from the GATS 2015, the prevalence of smoking was also lower in the healthcare facility sample – the prevalence ratio of current smoking was 0.77 (95% CI: 0.73–0.81).

### Selection and demographics of participants

**Fig. 1:**
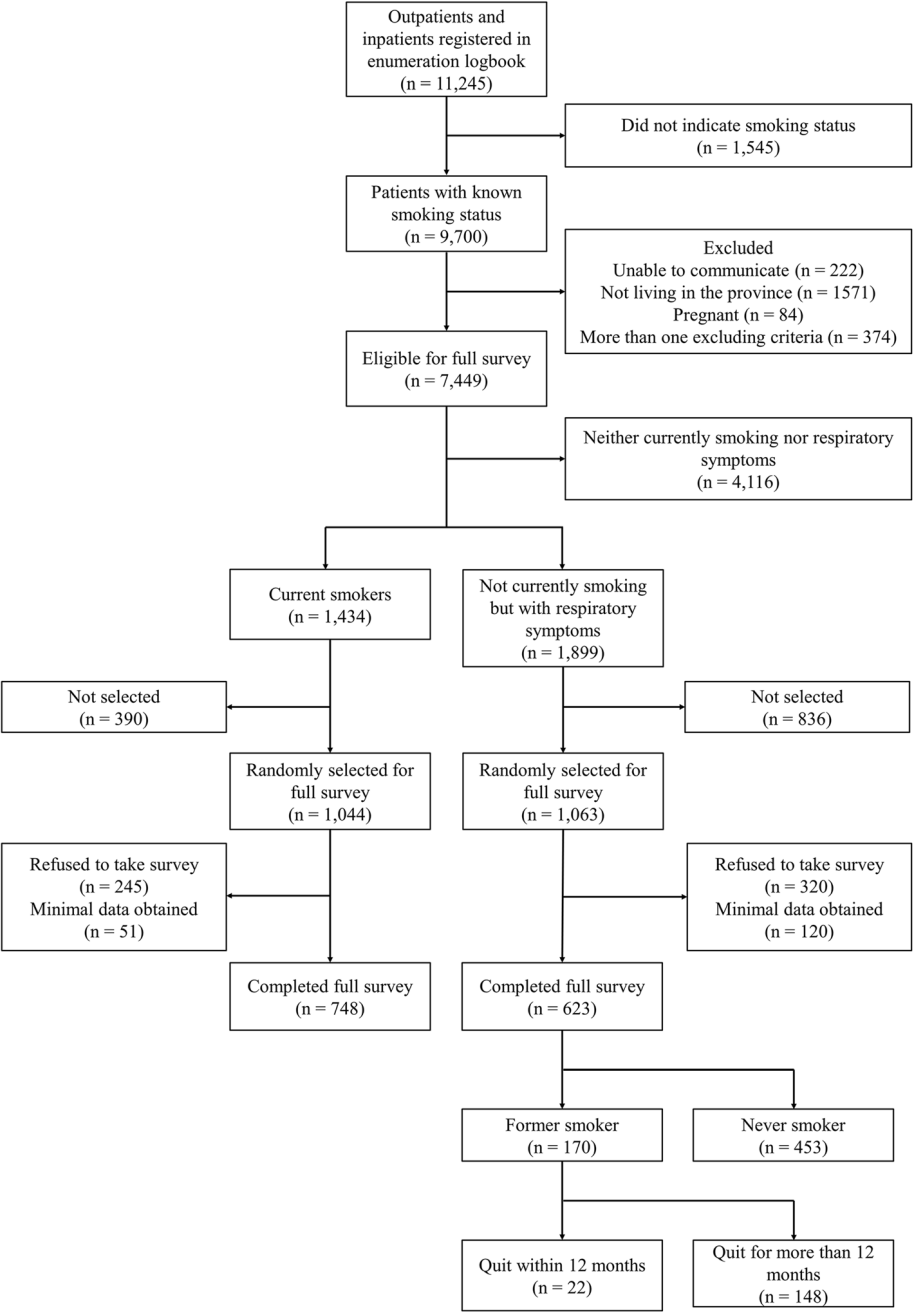
Consort diagram of participant recruitment

Figure 1 shows a flowchart of participant selection. A random sample of current smokers (1044 out of 1434 smokers) was selected to complete the full survey. Among these smokers, 748 (71.6%) completed the full survey. Among 623 participants who had respiratory symptoms but did not smoke, 170 were former smokers and 22 of them quit smoking within the past 12 months.

The majority (99.3%) of the 748 current smokers who completed the full survey were men. The median age was 57 years (interquartile range: 46–65). Approximately one in three (32.2%) current smokers lived with another smokers. In Additional file 1, Supplementary Table S1 shows the demographic characteristics of the 748 current smokers who completed the full survey. Supplementary Tables S2 and S3 compare the demographic characteristics of participants and non-participants who were current smokers. Supplementary Table S4 shows tobacco products used by the 748 current smokers.

### Smoking cessation attempts

Among 748 current smokers who completed the full survey, 254 (34%) reported having been asked if they smoked tobacco by a healthcare provider in the last 12 months, 494 (66%) reported having not been asked. During this time, 260 (34.8%) current smokers had tried to quit. Among the 254 patients who had been asked about smoking by a healthcare provider, 229 (90.2%) had been advised to quit by a healthcare provider and 103 (40.6%) had tried to stop smoking in the previous 12 months. Among the 494 patients who have not been asked about their smoking habits, 157 (31.8%) patients had tried to quit in the previous 12 months. Compared to current smokers who had not asked about smoking by a healthcare provider, those who had been asked had a higher chance of attempting to quit (40.6% vs 31.8%, *p* = 0.017).

Table 2 shows the proportion of participants who had used smoking cessation interventions among those who had tried to quit in the previous 12 months: including the 260 current smokers and 22 ex-smokers who had successfully quit within the past 12 months. The majority who had made quit attempts had done so without using any form of cessation assistance. Counselling had been used by 5 (1.9%) current smokers and nicotine replacement therapy had been used by 26 (10%) current smokers. Prescription medicines other than nicotine replacement therapy, traditional medicines, quit line, and smokeless tobacco had been used by less than 1% of current smokers. Among the 22 patients who successfully quit in the previous 12 months, only one reported having received counselling.

**Table 2:** Reported use of smoking cessation interventions among patients completing full survey who attempted to quit in the prior 12 months

	Currently smoking ***n*** = 260	Not currently smoking ***n*** = 22	Total ***n*** = 282
**Method of smoking cessation used in past 12 months (n, %)**^a^			
**Smoking cessation counselling**	5 (1.9)	1 (4.5)	6 (2.1)
**Nicotine replacement therapy**	26 (10.0)	0 (0)	26 (9.2)
**Other prescription medications (e.g. varenicline)**	2 (0.8)	0 (0)	2 (0.7)
**Traditional medicines**	1 (0.4)	0 (0)	1 (0.4)
**A quit line or a telephone support line**	1 (0.4)	0 (0)	1 (0.4)
**Use of smokeless tobacco**	1 (0.4)	0 (0)	1 (0.4)
**None of above methods used (n, %)**	230 (88.5)	21 (95.5)	251 (89.0)

^a^Patients may have used more than one method

### Stages of change

The stages of change among current smokers are shown in Table 3. When asked about readiness to quit, 116/632 (18.4%) current smokers wanted to quit within the next month. The proportion of patients wanting to quit in the next month was higher at central/provincial hospitals (71/234, 30.3%) than those visiting district hospitals (39/344, 11.3%, *p* < 0.001) and commune health centres (6/54, 11.1%, *p* = 0.004). Nevertheless, almost 40% of these current smokers did not consider quitting at all, with the proportion highest at commune health centres (25/54, 46.3%) and lowest at central/provincial facilities (78/234, 33.3%).

**Table 3:** Preparedness to quit smoking among current smokers completing full survey, by health system level^a^

	All facilities (46 facilities) ***n*** = 632	Central/provincial hospital (8 facilities) ***n*** = 234	District hospital (16 facilities) ***n*** = 344	Commune health centre (12 facilities) ***n*** = 54
**Plans to quit within the next month (n, %)**	116 (18.4%)	71 (30.3%)	39 (11.3%)	6 (11.1%)
**Plans to quit within the next 12 months (n, %)**	66 (10.4%)	28 (12.0%)	32 (9.3%)	6 (11.1%)
**Plans to quit someday, but not next 12 months (n, %)**	172 (27.2%)	45 (19.2%)	114 (33.1%)	13 (24.1%)
**Not currently interested in quitting (n, %)**	250 (39.6%)	78 (33.3%)	147 (42.7%)	25 (46.3%)
**Unknown/refused to answer (n, %)**	28 (4.5%)	12 (5.1%)	12 (3.5%)	4 (7.4%)

^a^116 missing values

## Discussion

This survey of patients from 46 health facilities in 4 provinces of Vietnam shows a high prevalence of smoking among male patients seeking healthcare. Current smokers who were asked about smoking by a healthcare provider were more likely to make quit attempts than those not asked. Smoking cessation aids and assistance were generally not used by smokers who attempted to quit. Current smokers visiting central/provincial hospitals were more inclined to quit, yet almost four in ten current smokers seeking healthcare were not interested in quitting smoking.

This study is the first to measure the prevalence of smoking among patients presenting to all four levels of Vietnam’s government healthcare system. Our finding on substantial sex difference is consistent with previously reported data in many low- and middle-income countries (LMICs) [15] and those collected among patients with HIV in Vietnam [16]. The higher prevalence among male patients aged 25 to 64 years is also in keeping with population-wide data [11]. Even though the high ratio of males to females among smokers in South East Asia and Western Pacific regions has been well documented, a recent scoping review found few research articles on the association between masculinity and smoking behaviour [17]. This association and effective interventions specifically for male smokers remain to be studied, especially in countries where male-to-female ratio of smoking prevalence is high.

Identifying patients who smoke by healthcare providers may increase the likelihood of quitting. A meta-analysis found that a system to screen tobacco use in healthcare settings significantly increases the chance of clinical intervention [18]. In our analysis, the majority of current smokers who had been asked about smoking behaviour also received advice to quit from healthcare providers. We also observed a higher proportion of attempting to quit among current smokers who had been asked about smoking by medical professionals than those who had not been asked. Nevertheless, only about one third of the current smokers in our study had been asked about their smoking behaviour in the past 12 months and a high proportion of current smokers did not want to quit. The findings warrant the implementation of screening for tobacco use and quit advice in healthcare facilities in Vietnam, particularly commune health centres where prevalence of current smoking is the highest.

After identifying smokers in healthcare settings, the establishment of other system-based approaches might increase the chance of quitting. This may include capacity building activities for healthcare workers, a reminder system to prompt cessation discussion with the patients [19], and incorporating cessation as a routine part of care management for patients admitted to hospitals [20, 21]. Optimal management for following up patients after discharge should be considered as well. The lower prevalence of current smoking in healthcare settings than in the general population, coupled with the finding that a third of current smokers lived with another smoker, suggests the importance of smoking cessation activities beyond the healthcare system. According to the GATS 2015, more than half of current smokers were considering quitting but less than one third of them ever visited to a healthcare provider during the previous 12 months [11]. An analysis from the same survey showed high secondhand smoke exposure in public places [22]. We agree with the recommendation from the GATS 2015 that the national cessation programme should be strengthened in order to better reach those smokers who do not access healthcare. A recent study showed a positive result about the toll-free quit line run by Bach Mai Hospital [23]. Currently, this quit line provides around 10 follow-up counselling calls over 12 months. Provision and promotion of similar quit line services to the entire country will benefit those who are not reached by healthcare-based interventions. Similarly, mobile phone-based tobacco cessation interventions (mCessation) may achieve effective and cost-effective results in Vietnam and other LMICs [24, 25]. A cluster randomised controlled trial evaluating the effectiveness of a smoking cessation intervention that incorporates mCessation is currently underway (registration number: ACTRN12620000649910). Other measures, such as community-based cessation interventions and implementation of smoke-free environment, may also increase smokers’ motivation to stop smoking. Another ongoing cluster randomised controlled trial attempted to assess the effectiveness of involving community health workers in smoking abstinence [26]. Further studies to evaluate the effectiveness and cost-effectiveness of different interventions, both healthcare-based and non-healthcare-based, are desirable.

We demonstrated a very low rate of utilisation of smoking cessation services among patients who made quit attempts in the past 12 months. This finding was similar to a cross-sectional survey among 321 men calling the quit line service run by Bach Mai Hospital [27]. Only less than 5% of these male smokers used direct counselling, nicotine replacement therapy, or medicines (bupropion/varenicline) before calling the quit line. An important barrier to accessing this service includes the lack of awareness of the phone number by smokers, which could be addressed by increasing funding for health promotion in Vietnam, and including the Quitline number on the packages of tobacco products [28].

Our analysis also showed differences in willingness to quit among patients at different levels of health facility. This finding, along with the differences in prevalence of smoking across sex, age groups, and levels of facility, indicates the need to tailor evidence-based smoking cessation interventions to the local context. An example to achieve this is the “Ottawa Model for Smoking Cessation”, a systematic approach to tobacco dependence management delivered for patients attending healthcare settings [21].

The strength of this study is inclusion of participants from all levels of the health system in four geographically distinct provinces of Vietnam, increasing its generalisability. We also used standardised questionnaires to assess current smoking behaviours, and contact with tobacco control services. However, our study sample may slightly under-represent the proportion of patients attending commune level facilities – in comparison to higher level facilities [29].

This study has a number of important policy implications. First, the low proportion of current smokers been asked about smoking habits highlights the need for a screening system to identify patients who smoke that can be integrated into routine practice. Second, the intervention to support quit smoking in the healthcare facilities should be tailored to patients’ characteristics and capacity of the facility. Third, even though cessation medications are effective in assisting smokers quit, these medications are expensive and not readily available in Vietnam. Policies to provide cessation medications covered by public health insurance that are cost-effective will be necessary to further reduce smoking prevalence.

Further research is required to address several questions. How smokers acquire information about cessation services and access assistance in Vietnam is still not clear. For example, the quit line operated by Bach Mai Hospital is the first national quit line service that has been available since 2015. It is desirable to know that smokers did not use this service because they were not aware of the service or they did not consider it helpful. A recent systematic review of randomised controlled trials showed that nicotine replacement therapy, behavioural counselling and brief advice are effective interventions in LMICs [30]. Nevertheless, implementing these interventions in healthcare settings remains a big challenge in many LMICs [31]. A flexible model to include evidence-based smoking cessation services into clinical practice in different levels of health facilities should also be established. Finally, it is needed to study the role of health authorities in supervising the implementation, which is critical to maintain the sustainability of the model.

## Conclusions

In conclusion, smoking is common among male patients presenting to healthcare facilities in Vietnam. Formal smoking cessation supports are rarely used by smokers attempting to quit. This is a population likely to benefit from a structured smoking cessation programme based on effective models of care.

## Supplementary Information

**Additional file 1: Supplementary Table S1.** Demographics of current smokers completing full baseline survey, by level of healthcare facility. **Supplementary Table S2.** Comparison between current smokers who were included and those who were not included at all healthcare facilities. **Supplementary Table S3.** Comparison between current smokers who completed the full survey, those who completed the minimal data questionnaire, and those who refused to participate at all healthcare facilities. **Supplementary Table S4.** Use of tobacco products among current smokers who completed the full survey

## Data Availability

The datasets used and/or analysed during the current study are available from the corresponding author on reasonable request.

## Abbreviations

WHO: World Health Organization
GATS: Global Adult Tobacco Survey
LMICs: Low- and middle-income countries
