# Supplementary material for: Smoking behaviour among adult patients presenting to health facilities in four provinces of Vietnam
Source: BMC Public Health. 2021 May 1;21:845. doi: 10.1186/s12889-021-10880-z (PMC8088640; doi:10.1186/s12889-021-10880-z)
Supplement: Supplementary file 1 — Additional file 1: Supplementary Table S1. Demographics of current smokers completing full baseline survey, by level of healthcare facility. Supplementary Table S2. Comparison between current smokers who were included and those who were not included at all healthcare facilities. Supplementary Table S3. Comparison between current smokers who completed the full survey, those who completed the minimal data questionnaire, and those who refused to participate at all healthcare facilities. Supplementary Table S4. Use of tobacco products among current smokers who completed the full survey [file 12889_2021_10880_MOESM1_ESM.pdf]

Supplementary Table S1. Demographics of current smokers completing full baseline survey, by level of healthcare facility

	All facilities (46 facilities) n = 748	Central/provincial hospital (8 facilities) n = 277	District hospital (16 facilities) n = 408	Commune health centre (12 facilities) n = 63
**Age, years (median, IQR)**	57 (46 - 65)	56 (42 - 64)	58 (48 - 65)	57 (48 - 65)
**Male gender (n, %)**	743 (99.3%)	276 (99.6%)	406 (99.5%)	61 (96.8%)
**Education level (n, %)**				
**Less than primary**	51 (6.8%)	18 (6.5%)	30 (7.4%)	3 (4.8%)
**Primary**	219 (29.3%)	89 (32.1%)	110 (27.0%)	20 (31.8%)
**Lower secondary**	283 (37.8%)	95 (34.3%)	161 (39.5%)	27 (42.9%)
**Upper secondary**	141 (18.9%)	49 (17.7%)	82 (20.1%)	10 (15.9%)
**University degree, or equivalent, or higher**	51 (6.8%)	24 (8.7%)	24 (5.9%)	3 (4.8%)
**Unknown/No answer**	3 (0.4%)	2 (0.7%)	1 (0.2%)	0 (0.0%)
**Occupation (n, %)**				
**Indoor manual labourer**	65 (8.7%)	24 (8.7%)	39 (9.6%)	2 (3.2%)
**Outdoor manual labourer**	68 (9.1%)	19 (6.9%)	45 (11.1%)	4 (6.3%)
**Agricultural work**	188 (25.1%)	69 (24.9%)	93 (22.9%)	26 (41.3%)
**Retired**	203 (27.1%)	72 (26.0%)	118 (29.0%)	12 (19.0%)
**Unemployed**	18 (2.4%)	5 (1.8%)	7 (1.7%)	6 (9.5%)
**Other**	206 (27.5%)	88 (31.8%)	105 (25.8%)	13 (20.6%)
**Comorbidity (n, %)**				
**Heart disease**	73 (9.8%)	25 (9.0%)	44 (10.8%)	4 (6.3%)
**Hypertension**	205 (27.4%)	58 (20.9%)	133 (32.6%)	14 (22.2%)
**Diabetes**	88 (11.8%)	21 (7.6%)	64 (15.7%)	3 (4.8%)
**Asthma**	45 (6.0%)	18 (6.5%)	19 (4.7%)	8 (12.7%)
**COPD**	30 (4.0%)	15 (5.4%)	14 (3.4%)	1 (1.6%)
**Chronic bronchitis**	67 (9.0%)	32 (11.6%)	25 (6.1%)	10 (15.9%)
**Emphysema***	3 (0.5%)	1 (0.4%)	2 (0.5%)	0 (0.0%)
**History of tuberculosis**	65 (8.7%)	38 (13.7%)	20 (4.9%)	7 (11.1%)
**Geographic area (n, %)**				
**Northern Vietnam**	389 (52.0%)	150 (54.2%)	205 (50.2%)	34 (54.0%)

**Hanoi**	169 (22.6%)	66 (23.8%)	103 (25.2%)	0 (0.0%)
**Thanh Hoa**	220 (29.4%)	84 (30.3%)	102 (25.0%)	34 (54.0%)
**Southern Vietnam**	359 (48.0%)	127 (45.8%)	203 (49.8%)	29 (46.0%)
**Ho Chi Minh City**	143 (19.1%)	36 (13.0%)	102 (25.0%)	5 (7.9%)
**Ca Mau**	216 (28.9%)	91 (32.9%)	101 (24.8%)	24 (38.1%)
**Had breathing problems that interfered with usual daily activities† (n, %)**	52 (7.7%)	18 (7.6%)	31 (8.0%)	3 (5.3%)
**Living with at least one other who smoked a cigarette, pipe or cigar in your home during the past two weeks (n, %)**	241 (32.2%)	92 (33.2%)	131 (32.1%)	18 (28.6%)

IQR: interquartile range; *83 missing values; †69 missing

Supplementary Table S2: Comparison between current smokers who were included and those who were not included at all healthcare facilities

	Eligible, not selected	Eligible, selected
Total	390	1044
Male (n, %)	367 (94.1%)	1,027 (98.4%)
Age, years (mean, SD)	52.7 (15.4)	53.9 (14.5)

Supplementary Table S3: Comparison between current smokers who completed the full survey, those who completed the minimal data questionnaire, and those who refused to participate at all healthcare facilities

	Full baseline survey	Minimal data	Refused to participate
Total	748	51	245
Male (n, %)	741 (99.1)	50 (98.0)	236 (96.3)
Age, years (mean, SD)	54.7 (14.4)	51.4 (13.2)	51.7 (14.7)

Supplementary Table S4: Use of tobacco products among current smokers who completed the full survey

	Current smokers<br>n = 748
Manufactured cigarettes (n, %)	550 (73.5%)
Hand-rolled cigarettes (n, %)	56 (7.5%)
Kreteks (n, %)	0 (0%)
Pipes full of tobacco (n, %)	0 (0%)
Cigars, cheroots, or cigarillos (n, %)	4 (0.5%)
Water pipes (n, %)	242 (32.4%)
Electronic cigarettes (n, %)	0 (0%)
